# Serpins: Genome-Wide Characterisation and Expression Analysis of the Serine Protease Inhibitor Family in *Triticum aestivum*

**DOI:** 10.1534/g3.119.400444

**Published:** 2019-06-21

**Authors:** Harriet R. Benbow, Lars S. Jermiin, Fiona M. Doohan

**Affiliations:** * School of Biology and Environment Science,; † Earth Institute,; ‡ Centre for Plant Science, University College Dublin, Belfield, Dublin 4, Ireland, and; § Research School of Biology, Australian National University, Canberra, ACT 2600, Australia

**Keywords:** Wheat, serine protease inhibitor, serpin, disease response, grain development

## Abstract

The serine protease inhibitor (serpin) gene family is the largest family of protease inhibitors. Serine protease inhibitors have an active, but under-characterized, role in grain development and defense against pathogen attack in cereal crops. By exploiting publicly available genomic, transcriptomic and proteomic data for wheat (*Triticum aestivum*), we have identified and annotated the entire ’serpinome’ of wheat and constructed a high-quality and robust phylogenetic tree of the gene family, identifying paralogous and homeologous clades from the hexaploid wheat genome, including the Serpin-Z group that have been well characterized in barley. Using publicly available RNAseq data (http://www.wheat-expression.com/), expression profiles of the wheat serpins were explored across a variety of tissues from the developing grain, spikelet and spike. We show that the *SERPIN-Z* clade, among others, are highly expressed during grain development, and that there is homeologous and paralogous functional redundancy in this gene family. Further to their role in grain development, serpins play an important but under-explored role in response to fungal pathogens. Using 13 RNAseq datasets of wheat tissues infected by fungal pathogens, we identified 37 serpins with a significant disease response. The majority of the disease-responsive serpins were upregulated by *Fusarium graminearum*, a destructive fungal pathogen that attacks the spike and developing grain of wheat. As serpins are ubiquitous in wheat grain, the genes encoding serpins may be linked to grain development, with their disease response a result of pleiotropy.

Food security remains one of the most important socioeconomic issues in the world. Bread wheat, *Triticum aestivum*, is one of the most important and widely grown food crops in the world (Shewry 2009). In fact, more land is dedicated globally to production of wheat (just under 250 million hectares in 2016) than to any other crop (http://www.fao.org/faostat, 2018). Although progress has been made in increasing yields through improved husbandry and breeding (Li *et al.* 2018a), over 37% of global wheat-producing areas are now facing yield stagnation, a problem that is almost ubiquitous across the highly-productive regions of Western Europe (Ray *et al.* 2012). Increases in potential wheat yields have dropped to just 1% per year (Fischer and Edmeades 2010), meaning that crop production, hindered by genetic limits on yield and rapidly-evolving pathogens, is failing to meet the demands of the growing population. Breeding targets remain focused around grain yield and disease resistance, with grain quality of near-equal importance (Guzman *et al.* 2016).

Vital for grain development (Østergaard *et al.* 2000) and implicated in disease response (Bao *et al.* 2018; Bhattacharjee *et al.* 2015; Bhattacharjee *et al.* 2017), serine protease inhibitor (serpin) genes make an interesting target for characterization and breeding. Serpins are the largest and most widely found family of protease inhibitors, found across the kingdoms Eukarya, Bacteria, Archaea and some viruses (Law *et al.* 2006; Rawlings *et al.* 2004), and are suicide inhibitors of serine protease enzymes (SPs) (Di Cera 2009). In general, serpin proteins are structurally well conserved, and composed of nine α-helices, three β-sheets, and a reactive central loop (RCL) that is exposed and mobile (Gooptu and Lomas 2009). The RCL acts as the substrate for the target protease and, when bound together, the serpin and the protease form a so-called Michealis complex (Huntington *et al.* 2000). Following formation of this complex, the P1-P1’ bond of the RCL is cleaved by the protease, prompting the conformational change of the serpin to a relaxed state from its native stressed state, as the RCL inserts itself between the β-sheets (Andersen *et al.* 2017; Stratikos and Gettins 1999). This action pulls the protease down to the lower pole of the serpin, and distorts the catalytic triad of the protease, rendering it inactive (Huntington *et al.* 2000).

Based on studies from wheat and its cereal relatives, it is evident that the serpin gene family is pivotal for grain development and quality. A review by Roberts and Hejgaard (2008) describes how more than 20 serpin genes have been cloned from barley, wheat, rye and oats, following the discovery of a barley grain serpin that acts as a storage protein during grain filling, and contributes a substantial part of the lysine (an essential amino acid in the human diet) content in barley grains (Hejgaard *et al.* 1985; Roberts and Hejgaard 2008). More recently, six serpins were identified in the wheat grain that show inhibitory activity toward the proteases chymotrypsin and cathepsin (Østergaard *et al.* 2000). The authors propose that these serpins may have evolved to inhibit these endogenous or exogenous proteases from breaking down vital grain storage proteins. Supporting this theory is the evidence that these wheat grain serpins are specific to proteases that have an affinity for proteins rich in proline and glutamine residues, such as prolamine – the protein that provides half of total grain nitrogen and the low and high molecular weight subunits of glutenin – vital for bread making (Østergaard *et al.* 2000; Bonnot *et al.* 2015).

Serpins also play an important role in defense against plant pathogens and thus have potential as breeding targets to improve disease resistance in wheat. Serpins have been investigated for their role: in defense response in plants such as soybean (Solomon *et al.* 1999), maize (Erb *et al.* 2009), and *Arabidopsis thaliana* against the necrotrophic fungus *Botrytis cinerea* (Laluk and Mengiste 2011), in programmed cell death (Fluhr *et al.* 2012), and as a regulator of effector-triggered immunity (Bhattacharjee *et al.* 2017), an integral part of the plant disease response. In tomato, *Solanum lycopersicum*, serine proteases secreted by *Fusarium oxysporum* (in response to chitinase attack by the plant) were inhibited by tomato serpins (Jashni *et al.* 2015), demonstrating that the chitinase-serine protease-serpin relationship is an important part of the plant defense to fungal pathogens. Some proteases secreted from cereal fungal pathogens have also been shown to act as pathogen effectors; subtilisin and trypsin-like proteases from *Fusarium culmorum* interact with barley protease inhibitors (Pekkarinen *et al.* 2007), proteases with signal peptides from *Fusarium graminearum* are secreted *in planta* but not *in vitro* (Paper *et al.* 2007), and a small protease is secreted by *Zymoseptoria tritici* during infection of wheat (Mirzadi Gohari *et al.* 2015).

In terms of wheat fungal pathogens, the causal agent of Septoria tritici blotch, *Zymoseptoria tritici*, displays high protease activity during early infections, and up to 11 days post application of the fungus on the wheat leaf (Palma‐Guerrero *et al.* 2015; Sánchez-Vallet *et al.* 2015). A serpin gene has been found to be responsive to *Z. tritici* during the first 12 days of infection (Adhikari *et al.* 2007). *Fusarium culmorum*, a causal agent of *Fusarium* head blight (FHB) disease, has well-studied protease activity linked to grain damage and yield loss in wheat (Wang *et al.* 2005). Five serpin genes have been found to be upregulated in a FHB-resistant wheat cultivar in response to *Fusarium* infection (Gottwald *et al.* 2012). Serpins are also likely to play a role in the wheat response to other pathogens, given the expression profiles of pathogen proteases: for example, in *Puccinia striiformis*, which causes stripe rust in wheat, 7.6% of the fungal secretome was made up of proteases (Xia *et al.* 2017). In other cereal crops, serpins have also been implicated in disease resistance, including resistance to FHB disease of barley caused by *F. culmorum* (Pekkarinen *et al.* 2007), *Rhizoctonia solani*-induced cell death in rice (Bhattacharjee *et al.* 2015), and resistance to rice blast, caused by *Magnaporthe oryzae* (Quilis *et al.* 2014).

In this study, we exploit the available sequence and expression data for wheat to test our hypotheses that serpin genes are involved in grain development and disease resistance in hexaploid bread wheat. Traditionally, the complexity of the large (∼16 Gbp), transposable element-rich, repetitive, allohexaploid wheat genome (Choulet *et al.* 2010; IWGSC 2014; Appels *et al.* 2018) made gene discovery and characterization difficult. Wheat contains three highly similar sub-genomes (A, B and D), each derived from three ancestral grass species (Shewry 2009) and they show high collinearity and sequence conservation between homeologous (homologous genes resulting from allopolyploidy (Glover *et al.* 2016)) sets of genes (Appels *et al.* 2018). A high-quality reference sequence for wheat (IWGSC RefSeq v1.0) has been published (Appels *et al.* 2018), bringing with it a comprehensive analysis of genome composition and a thorough dissection of wheat transcription across tissues, homeologues, and conditions (Ramírez-González *et al.* 2018). The collation of this data and the development of a wheat expression platform (Borrill *et al.* 2016; Ramírez-González *et al.* 2018) provides an extensive resource for hypothesis testing without the need for undertaking RNAseq analysis *de novo*. Using this resource, we present evidence that serpin genes play a role in resistance to fungal diseases in wheat, supplementing their utility in grain development.

## Materials and Methods

### Reference protein annotations

The wheat protein annotation from IWGSC RefSeq v1.0 (Appels *et al.* 2018) was downloaded from the IWGSC sequence repository hosted by URGI (https://urgi.versailles.inra.fr/download/iwgsc/IWGSC_RefSeq_Assemblies/v1.0/, accessed December 2017). High- and low-confidence annotations were concatenated into one FASTA file, which was used for all subsequent analysis.

### Serpin identification

A Hidden Markov Model (HMM) profile for the serpin protein family (PFAM: PF00079, InterPro: IPR023796) was retrieved from Pfam. The HMM profile was used to search the protein annotation using HMMER (http://hmmer.org/), with an E-value threshold of 1e^-5^. All protein sequences identified by the HMMER analysis were retrieved from the protein annotation using SAMtools fasta index (Li 2011). All BLAST searches (Altschul *et al.* 1990) were performed locally using BLAST+ (Camacho *et al.* 2009). Wheat serpin paralogues and homeologues, and serpin orthologs in *Arabidopsis thaliana*, *Brachypodium distachyon*, *Oryza sativa* and *Hordeum vulgare* were accessed through EnsemblPlants Biomart (http://plants.ensembl.org/Triticum_aestivum/Info/Index, 2019).

### Alignment and phylogenetic analysis

Multiple sequence alignment (MSA) was performed with the Clustal Omega (Sievers *et al.* 2011) using the R Bioconductor package “msa” (Bodenhofer *et al.* 2015). Completeness scores for the alignments were generated with AliStat v1.7 (Wong *et al.* 2019) and all sites in the alignments with a *C_c_* score (*C_c_* = number of unambiguous characters in a column of an MSA / number of sequences) below 0.4 were masked (*i.e.*, excluded from further analysis). For each pair of sequences for which *C_ij_* (*C_ij_* = number of columns in an MSA where the corresponding characters of both *i*- and *j*-th sequence are unambiguous / length of alignment) was 0, the sequence with the lowest *C_r_* score (*C_r_* = number of unambiguous characters in the sequence / alignment length) was removed and the alignment was re-inferred with the new subset of sequences. A matched-pairs test of symmetry, implemented in Homo v1.3 (Rouse *et al.* 2013), was used to test whether any pairs of sequences violate the phylogenetic assumption of evolution under stationary or reversible conditions (Ababneh *et al.* 2006). ModelFinder (Kalyaanamoorthy *et al.* 2017) was used to identify the best-fitting model of sequence evolution (SE) that led to the sequences. The model of SE with the lowest Bayesian information criterion (BIC) was chosen, and a phylogenetic tree was inferred using IQ-TREE (Nguyen *et al.* 2015) assuming the optimal model of SE. Tree branch support was estimated with 1000 ultrafast bootstrap replicates (UFBoot2) (Hoang *et al.* 2018).

### Expression analysis – biotic stress

Transcript count data for datasets relating to wheat-fungal pathogen interactions were downloaded from the available wheat RNAseq datasets on expVIP (Borrill *et al.* 2016; Ramírez-González *et al.* 2018; Islam *et al*. 2016; Rudd *et al*. 2015; Ma *et al*. 2014; Powell *et al*. 2017; Schweiger *et al*. 2016; Kugler *et al*. 2013; Yang *et al*. 2013; Zhang *et al*. 2014). Differential expression testing was conducted using DESeq2 (Love *et al.* 2014). When the data contained multiple cultivars or time-points, differential expression was conducted between treated and control samples per cultivar per time-point. Fold change of transcript abundance was expressed as log_2_ fold change, and a false-discovery rate (FDR) threshold of 0.05 (Benjamini and Hochberg 1995) was applied to correct the *P*-values for multiple comparison testing. Following differential expression analysis, the number of differentially expressed genes from each dataset was counted, and any datasets that were outliers (in terms of the number of differentially expressed genes they produced) were removed. The datasets retained and used for the remainder of the analysis are described in Table S1.

### Expression analysis – grain development

Normalized expression data (in transcripts per million (TPM)) were retrieved from expVIP for all tissues pertaining to the grain, spikelet or wheat head (Li *et al*. 2013; Pearce *et al*. 2015; Gillies *et al*. 2012; Pfeifer *et al*. 2014; Barrero *et al*. 2015) (Table S2). Low abundance filtering was carried out using TPM < 0.5 as a cut-off, as per Ramírez-González *et al.* (2018).

All statistical analyses were performed in R version 3.5.1.

### Data availability

Details of the RNAseq studies used are available in tables S1 and S2. Protein sequence alignments are available in fasta format in supplemental files 1-3. File S1 contains the original alignment of all 189 serpin proteins. File S2 contains the alignment of all serpin proteins, with uninformative sites masked. File S3 is the final alignment used to build the phylogenetic tree, with uninformative sites masked, and sequences that had no overlap removed. Supplemental material available at FigShare: https://doi.org/10.25387/g3.7910417.

## Results

### Serpin discovery

Using the HMM profile of plant serine protease inhibitors, 189 putative serpins were retrieved from the IWGSC RefSeq v1.0 protein annotation (containing 269,472 sequences). To pass the inclusion threshold, the entire sequence or, if incomplete, the best domain had to align to the HMM profile with e < 0.05. E-values ranged from 0.0036 – 2.5e^-106^. From the 189 putative serpins, 64 came from the IWGSC low-confidence annotation, with the remaining 125 being high-confidence annotations. Serpin genes (high- and low-confidence) were distributed unequally across the chromosomes, with Chromosome Groups 2, 4 and 5 having the highest density of serpins (Table 1). The 189 putative serpins from wheat were used as query terms, and orthologs for other plant species of interest were downloaded via EnsemblPlants Biomart. The number of orthologous serpin genes in the *A. thaliana* genome was 20, a 9.5 fold decrease compared to wheat. The *B. distachyon*, *H. vulgare* and *O. sativa* genomes housed 80 serpins (2.3 fold decrease), 51 serpins (3.7 fold decrease), and 83 serpins (2.3 fold decrease), respectively.

**Table 1:** The number of wheat serine protease inhibitor (serpin) genes found on each chromosome of the wheat genome

	Subgenome*			
Chromosome	A	B	D	U
1	3	2	3	—
2	10	15	15	—
3	9	10	6	—
4	14	12	7	—
5	13	18	11	—
6	6	11	6	—
7	5	2	7	—
U	—	—	—	4

* Serpin genes were mined from the wheat reference gene annotations (IWGSC v1.0) using a hidden markov model profile of the serpin protein family. 189 serpin genes were found, 4 of which were unassigned to a chromosome (U), and the remainder were assigned to the A, B or D wheat subgenomes.

### Alignment and phylogenetic analysis

The original alignment (supplementary file 1) contained sites and sequences with low completeness scores (*i.e.*, *C_c_* < 0.4 and *C_ij_* = 0), and the overall completeness score for the alignment was 0.19 (out of 1). Every site in the alignment with a completeness score < 0.4 was masked to remove any uninformative, low-complexity sites (that subsequently do not add much, if any, information to the phylogenetic result), and the completeness score for the masked alignment (supplementary file 2) increased to 0.7. As a result of iteratively removing sequences to avoid pairs of sequences that had no overlap, 50 sequences were removed, and the final, high-quality alignment (supplementary file 3) consisted of 139 sequences and 375 sites. The completeness score for this final alignment was 0.85, indicating a high-quality and potentially very informative alignment. Based on the matched-pairs test of symmetry, we discovered no evidence of violation of the phylogenetic assumption of evolution under reversible conditions.

**Figure 1:**
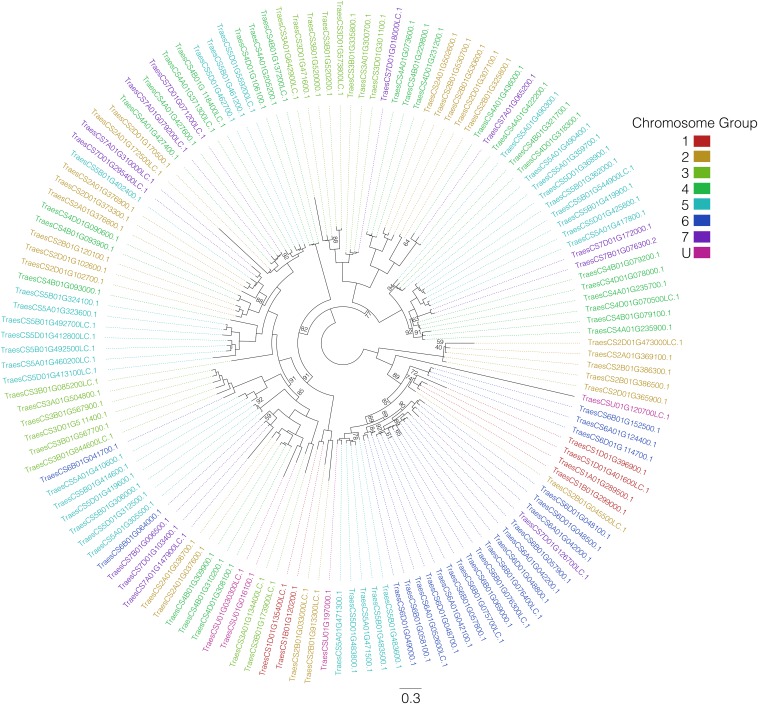
A maximum likelihood phylogenetic tree of wheat serpin proteins. Phylogeny was created using the JTT-R5 substitution model and branch length was supported with 1000 bootstraps and bootstrap support values below 95 are indicated on the tree (all other values are 95% or above). Font color represents chromosome group. In general, serpins cluster in homeologues or paralogues (or both), with low substitution rate within groups.

The optimal model of SE for these data were found to be the JTT model (Jones *et al.* 1992) with rate-heterogeneity across sites modeled using the PDF model with five categories (Kalyaanamoorthy *et al.* 2017). The total tree length (sum of branch lengths) was 30.4, implying that the final alignment contains plenty of information for phylogenetic inference, and average bootstrap branch support was 95.12, implying that this information contains a mostly consistent signal. In general, genes clustered together (Figure 1), and in some cases these clusters contained more than one set of homeologous genes that were paralogous.

### Response to disease-causing Fungi

To identify serpins upregulated in response to exposure to fungal pathogens, differential expression analysis was carried out using publicly available datasets of wheat treated with the fungal pathogens *Puccinia striiformis*, *Zymoseptoria tritici*, *Blumeria graminis*, *Fusarium pseudograminearum*, *Fusarium graminearum*, *Magnaporthe oryzae*, and the pathogen associated molecular pattern (PAMP) elicitors chitin and flg22. Serpin genes were extracted from the differentially expressed genes. In total, 20 (10.5% of the gene family) serpin genes were differentially expressed (FDR < 0.05; -1 ≥ Log_2_ Fold Change ≥1) in response to one or more of *F. graminearum*, *F. pseudograminearum*, *P. striiformis*, *Z. tritici*, chitin or Flg22. Fourteen serpin genes were up-regulated in response to fungal stress, four were down-regulated, and two were up-regulated by one agent and down by another (Table 2).

**Table 2:** Log_2_-fold change of serpin genes in response to fungal pathogens or PAMP elicitors

Serpin gene	Fungal pathogen*					PAMP elicitor	
	*Bg*	*Fg*	*Fp*	*Ps*	*Zt*	Flg22	Chitin
TraesCS2B01G033100LC	—	4.76	—	—	—	—	—
TraesCS2B01G033300LC	—	3.86	—	—	—	—	—
TraesCS2B01G530600	−3.08	1.01	—	—	—	—	—
TraesCS3B01G335800	—	2.94	—	—	—	—	—
TraesCS3D01G301100	—	1.92	—	—	—	—	—
TraesCS4A01G205200	—	—	—	—	—	2.41	—
TraesCS4A01G235700	−1.29	—	—	−2.26	1.04	—	—
TraesCS4A01G422200	—	3.56	—	—	—	—	—
TraesCS4A01G436000	—	1.56	—	—	—	—	—
TraesCS4B01G079100	—	—	−1.71	—	—	—	—
TraesCS4B01G079200	—	—	—	−1.03	—	—	—
TraesCS4D01G090600	—	—	—	—	—	4.43	5.07
TraesCS4D01G106100	—	1.21	—	—	—	—	—
TraesCS4D01G231200	−2.32	—	—	−1.27	—	—	—
TraesCS5B01G402400	—	—	—	—	—	—	4.30
TraesCS5B01G492700LC	—	—	—	—	—	−2.86	—
TraesCS6B01G068900	—	1.10	—	—	—	—	—
TraesCS6B01G152500	—	—	—	—	—	1.74	1.90
TraesCS6D01G048700	—	—	—	—	—	—	2.24
TraesCS6D01G114700	—	—	—	—	—	1.51	1.40

* Bg = Blumeria graminis (powdery mildew).

Fg = *Fusarium* graminearum (*Fusarium* head blight).

Fp = *Fusarium* pseudograminearum (*Fusarium* crown rot).

Ps = Puccinia striiformis (Stripe rust).

Zt = Zymoseptoria tritici (Septoria tritici blotch).

- = Non-significant fold change.

In general, the genes were responsive to one biotic agent; however, six of the 20 showed a broader response to fungal disease. Serpin TraesCS4A01G235700 was down-regulated by *Blumeria graminis* and *Puccinia striiformis* but upregulated by *Zymoseptoria tritici*. TraesCS2B01G530600 was down-regulated by *B. graminis* and upregulated by *F. graminearum*, and TraesCS4D01G231200 was down-regulated by both *B. graminis* and *P. striiformis*. TraesCS4D01G090600, TraesCS6B01G152500 and TraesCS6D01G114700 were each upregulated by both the PAMP elicitors, chitin and Flg22. The pathogen-responsive serpin genes were located on chromosomes 2B, 3B, 3D, 4A, 4B, 4D, 5B, 6B and 6D. Within these disease responsive genes, there were four pairs of genes that were homeologues of each other (Table 3). A pair of homeologous genes from the Group 3 chromosomes (B and D copies) were both responsive to *F. graminearum*, but the A-genome homeologue of this group was not responsive to any of the biotic stresses. Two pairs of homeologous genes from the Group 4 chromosomes were differentially expressed in response to biotic agents. TraesCS4A02G205200 and its D-genome homeologue, TraesCS4D02G106100, were upregulated by Flg22 and *F. graminearum*, respectively. TraesCS4A02G235700 was down-regulated by *B. graminis* and *P. striiformis* but upregulated by *Z. tritici*, while its B-genome counterpart was only down-regulated by *B. graminis*. Two genes from the Group 6 chromosomes, TraesCS6B02G152500 and TraesCS6D02G114700, from the B and D genomes, respectively, had very similar expression profiles, both being upregulated in response to the PAMP-elicitors chitin and Flg22.

**Table 3:** Homeologous pairs of serpins that are responsive to one or more pathogen

Homeologous group	Gene	Log_2_ fold change	Stress
1	TraesCS3B1G335800	2.94	*F. graminearum*
	TraesCS3D01G301100	1.92	*F. graminearum*
2	TraesCS4A01G205200	2.41	Flg22
	TraesCS4D01G106100	1.21	*F. graminearum*
3	TraesCS4A01G235700	−1.29	*B. graminis*
	TraesCS4A01G235700	−2.26	*P. striiformis*
	TraesCS4A01G235700	1.04	*Z. tritici*
	TraesCS4B01G079200	−1.03	*P. striiformis*
4	TraesCS6B01G152500	1.9	Chitin
	TraesCS6B01G152500	1.74	Flg22
	TraesCS6D01G114700	1.4	Chitin
	TraesCS6D01G114700	1.51	Flg22

Nine of 21 of the disease-responsive serpins were responsive to *F. graminearum* infection. These 9 serpins were up-regulated by *F. graminearum* across both resistant and susceptible wheat that were near isogenic lines (NILs) for FHB resistance quantitative trait loci (QTL) (Schweiger *et al.* 2016). Eight of these nine *Fusarium* responsive serpins were up-regulated at 48 hr post inoculation of the wheat heads with *F. graminearum*, three were upregulated at 36 hr, and two at 24 hr, although only in the susceptible NIL (the line carrying susceptible alleles for FHB-resistance QTL *Fhb1* and *Qhfs.ifa-5A* (Schweiger *et al.* 2016) (Figure S1).

**Figure 2:**
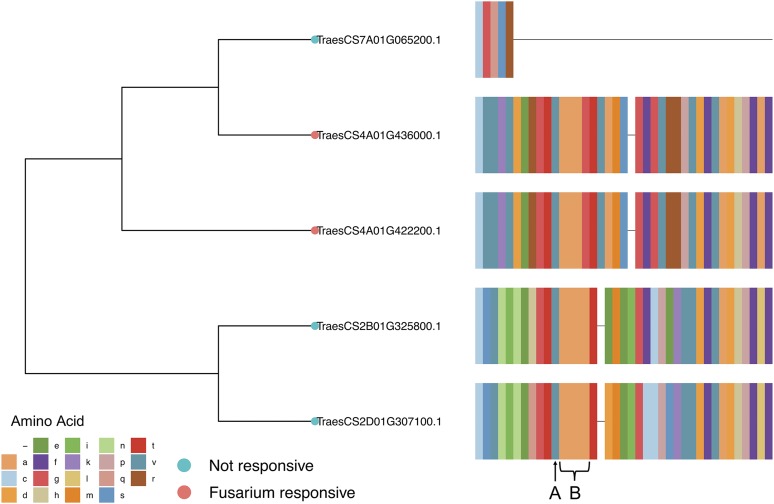
Multiple protein sequence alignment of the hinge region and reactive central loop (RCL) of five serpins from the same subclade. Two paralogous serpin genes from chromosome 4A are up-regulated by *Fusarium graminearum* infection, whereas the closest paralogues of these genes are not. The RCLs of the *Fusarium* responsive serpins are identical and are different to those of the non-*Fusarium* responsive serpins. The RCL regions starts at P17, with a glycine residue (A), followed by a well conserved alanine-rich region (B).

In general, the biotic stress-responsive serpins do not cluster in phylogenetic clades but are dispersed throughout the tree of serpins. However, five out of the nine serpins that are responsive to *F. graminearum* are specific to one subclade, containing 20 genes (Figure S2). Of these five genes, there are two pairs of paralogous and homeologous genes. TraesCS4A01G436000 and TraesCS4A01G422200 are *Fusarium*-responsive paralogues with 80% of the sites in the protein sequences of these genes being identical. These genes are located within a clade containing three other serpins. The nearest phylogenetic neighbor of the chromosome 4A serpins, TraesCS7A01G065200, is not *Fusarium* responsive, and only shares 65% and 77% similarity with the genes on Chromosome 4A, respectively. Also within this sub-clade are two genes embedded in Chromosomes 2B and 2D. These two serpins are also not responsive to *Fusarium*. The hinge region and the RCL of the Chromosomes 4A *Fusarium*-responsive serpins have identical sequences, compared to the non-*Fusarium*-responsive serpins within this clade (Figure 2).

### Serpin expression in the wheat grain

**Figure 3:**
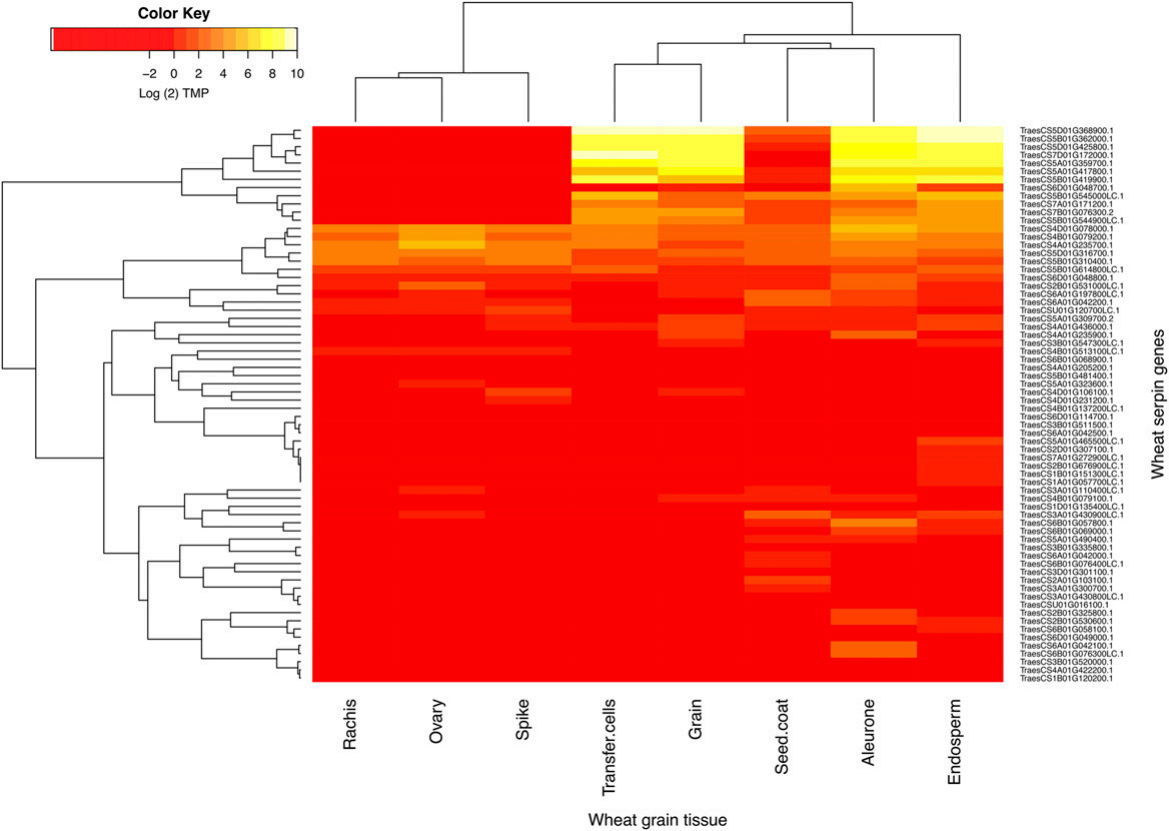
Expression of wheat serpin genes in various tissues of the wheat head or developing grain. The heatmap displays expression, as Log_2_ (transcripts per million (TPM)) of each gene across seven tissues of the developing grain of wheat. Red denotes low levels of expression, with high expression being displayed in yellow. The serpin genes with the highest expression in the grain are the *SERPIN-Z* genes, a group of storage proteins that are important in grain development. These genes are located on the group 5 chromosomes of wheat.

The transcriptional profiles of the serpins were assessed across seven RNAseq datasets detailing the wheat spike, rachis, whole grain, or specific grain tissues (aleurone, endosperm, ovary, seed coat and transfer cells) across a variety of timepoints and cultivars. In total, there were 31 combinations of tissues and timepoints across the datasets. The data dimensions were reduced by grouping timepoints together and categorizing samples by tissue: aleurone, endosperm, grain, ovary, rachis, seed coat, spike, and transfer cells. Of the 189 serpins, 55 (29%) were expressed in one of more of the datasets above the threshold of 0.5 TPM. The serpins were found across various spike and grain tissues, including the rachis and spike itself, but were most abundant in the aleurone and endosperm (Figure 3).

**Figure 4:**
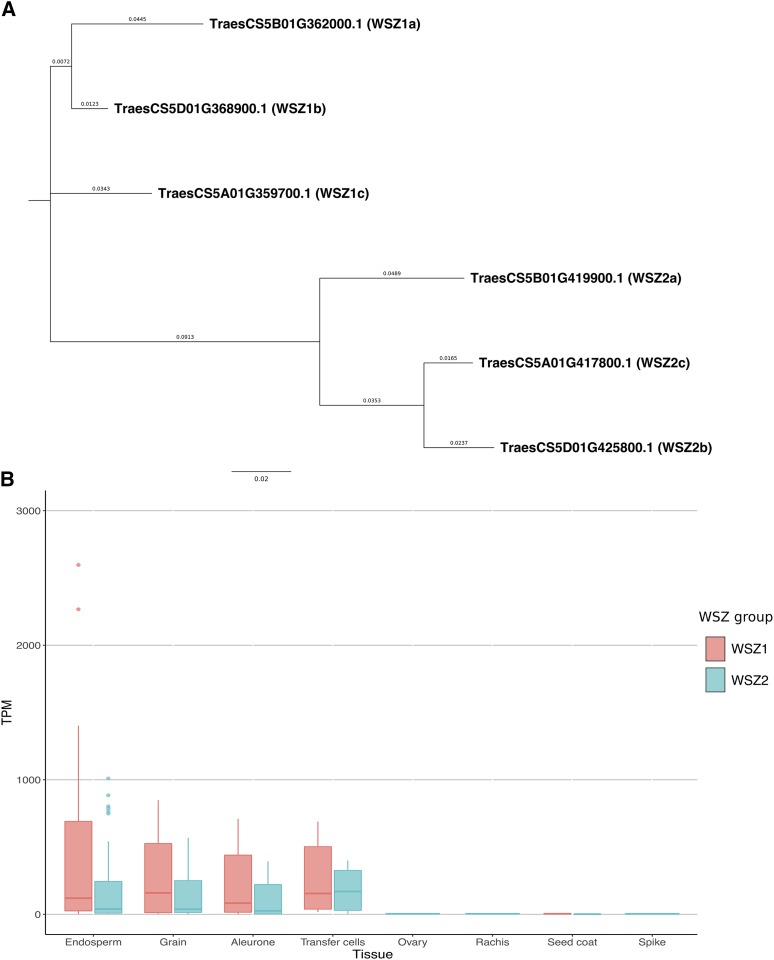
The SERPIN-Z proteins in wheat consist of two groups (1 and 2) of homeologous genes. (A) The six SERPIN-Z proteins form two phylogenetic groups, with each group comprising three members, from the A, B and D genome. (B) Expression of *SERPIN-Z* genes in transcripts per million (TPM) across spike and grain tissues. “WSZ group” refers to the SERPIN-Z group of storage proteins, which can be separated into two groups, Z1 and Z2. These genes are highly expressed in the endosperm, whole grain, aleurone and transfer cells of the developing wheat grain, and expressed at very low levels in the ovary, rachis, seed coat or whole spike. Overall, the *SERPIN-Z1* group of genes were expressed at a higher level than the *Z2* group.

The serpins with the highest expression across the grain tissues were identified as the wheat SERPIN-Z family by a BlastP search of serpins against the UniProt protein sequence for wheat serpins WSZ1a, WSZ1b, WSZ1c, WSZ2a and WSZ2b. The SERPIN-Z clade consists of two homeologous sets of serpins from the Group 5 chromosomes, group 1 and group 2, for which gene expression across grain tissues was high (Figure 4). Expression of the group 1 *SERPIN-Z* genes was higher than that of the group 2 *SERPIN-Z* genes.

By comparing amino acid sequence data of serpins WSZ1a, WSZ1b, WSZ1c, WSZ2a and WSZ2b from Østergaard *et al.* (2000), who sequenced the active site of these WSZ proteins, 5 of the IWGSC wheat proteins were unambiguously identified as the *WSZ* genes 1a, 1b, 1c, 2a, and 2b. Østergaard *et al.* (2000) only provide sequence information for 5 SERPIN-Z proteins, however our study identified 6 high-confidence protein annotations that were putative serpin-Z genes. Rosenkrands *et al.* (1994) partially sequenced a wheat SERPIN-Z protein (called WSZ at the time) from the wheat grain, which matches the amino acid sequence of the 6^th^ putative serpin protein annotation (Table 4). Therefore, we suggest that this protein is WSZ2c, the A-genome homeologue of WSZ2a and WSZ2b.

**Table 4:** Wheat SERPIN-Z proteins. The amino acids that were previously confirmed by partial sequencing of SERPIN-Z WSZ1a, WSZ1b, WSZ1c, WSZ2a and WSZ2b (Østergaard *et al.* 2000) and WSZ2c (previously called WSZ (Rosenkrands *et al.* 1994)) are in bold, and the active site residues at P1 and P1’ (the site of cleavage in the reactive central loop) are underlined. The sequenced amino acids match with 100% identity to the corresponding sites in the IWGSC protein annotation

IWGSC ID	Serpin Z protein	Active site sequence
TraesCS5B01G362000	WSZ1a	-K**MVLQQ**ARPPS-
TraesCS5D01G368900	WSZ1b	-K**MVPQQ**ARPPS-
TraesCS5A01G359700	WSZ1c	-K**MALLQ**ARPPS-
TraesCS5B01G419900	WSZ2a	-K**AVLLS**ASPPS-
TraesCS5D01G425800	WSZ2b	-KVVLR**QAPPP**S-
TraesCS5A01G417800	WSZ2c	-KAVLR**QARPPS**-

Across the 31 conditions, 54 serpins were expressed in more than one dataset, and four serpins were expressed ubiquitously across all sets. Of these four genes, three were homeologues of each other from the Group 4 chromosomes; TraesCS4A01G235700, TraesCS4B01G079200 and TraesCS4D01G078000. TraesCS5D01G316700 was also expressed across all tissue × timepoint × cultivar combinations, but as a singular gene, not a homeologous triad.

### Pleiotropy in the serpin family

**Figure 5:**
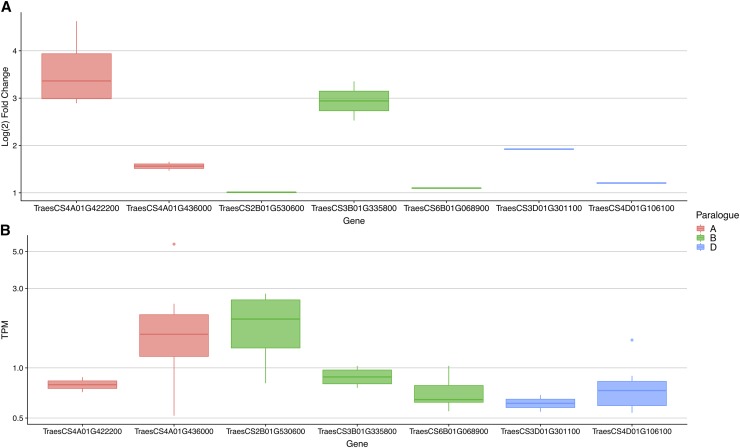
Pleiotropic serpin genes are up-regulated by *Fusarium graminearum* and expressed in the developing grain. (**A**) Seven serpin genes are up-regulated by the head blight pathogen *F. graminearum*. Y-axis values are Log_2_ fold change of the gene between wheat heads treated with *F. graminearum* or a mock solution, across two wheat genotypes and 3 timepoints. (B) The same seven serpins are also expressed in the developing grain of wheat. Y-axis values are transcripts per million, and the y-axis is log_10_ transformed. The seven pleiotropic serpins belong to three distinct paralogous groups, grouped by color.

In total, 20 serpins were responsive to biotic stressors, 55 were expressed in one or more tissues of the wheat grain, and 14 were both disease responsive and expressed in the developing grain (Table 5). Seven of these pleiotropic serpins were responsive to foliar pathogens, chitin and Flg22. With the exception of *Z. tritici*, chitin and Flg22, these serpins were down-regulated by fungal pathogens that do not attack the wheat head. Unsurprisingly, given that FHB is a disease of the wheat head, seven of the 14 pleiotropic serpins were responsive to the head-blight pathogen *F. graminearum*; six were responsive to only *F. graminearum* and one (TraesCS2B01G530600) was up-regulated by *F. graminearum* and down-regulated by *B. graminis*. The *F. graminearum*-responsive serpins belonged to three paralogous groups based on the paralogue data downloaded from EnsemblPlants (Figure 5), and within this set, the genes TraesCS3B01G335800 and TraesCS3D01G301100 were homeologues.

**Table 5:** Pleiotropic serpin genes are responsive to biotic stress and expressed in the developing grain of wheat

		Stress**							Grain and spike							
Group*	Serpin	*Bg*	Chitin	*Fg*	Flg22	*Fp*	*Ps*	*Zt*	Aleurone	Endosperm	Ovary	Rachis	Seed coat	Spike	Transfer cells	Whole grain
1	TraesCS3D01G301100	—	—	1.92	—	—	—	—	—	0.69	—	—	0.55	—	—	—
	TraesCS3B01G335800	—	—	2.94	—	—	—	—	0.82	0.76	—	—	1.01	—	—	—
2	TraesCS4D01G106100	—	—	1.21	—	—	—	—	—	0.66	0.69	—	—	2.32	—	1.12
	TraesCS4A01G205200	—	—	—	2.41	—	—	—	—	0.61	—	—	—	0.76	—	—
3	TraesCS4B01G079200	—	—	—	—	—	−1.03	—	19.34	11.76	17.01	4.54	8.19	5.1	11.01	5.94
	TraesCS4A01G235700	−1.29	—	—	—	—	−2.26	1.04	15.02	12.2	39.53	9.86	7.3	5.67	9.26	3.56
4	TraesCS2B01G530600	−3.08	—	1.01	—	—	—	—	1.27	1.55	—	—	—	—	—	—
5	TraesCS4A01G436000	—	—	1.56	—	—	—	—	1.92	1.73	—	—	1.21	1.05	1.7	2.13
6	TraesCS4A01G422200	—	—	3.56	—	—	—	—	0.8	—	—	—	—	—	—	—
7	TraesCS4B01G079100	—	—	—	—	−1.71	—	—	1.67	1.67	—	—	0.87	—	—	1.14
8	TraesCS4D01G231200	−2.32	—	—	—	—	−1.27	—	—	0.51	—	—	—	1.05	—	0.62
9	TraesCS6B01G068900	—	—	1.1	—	—	—	—	0.79	0.78	0.63	—	—	0.62	—	—
10	TraesCS6D01G048700	—	2.24	—	—	—	—	—	22.87	3.11	—	—	0.97	—	0.5	1.09
11	TraesCS6D01G114700	—	1.4	—	1.51	—	—	—	—	0.58	—	—	—	—	—	—

* Group refers to homeologous group. Groups 1-3 each contain 2 serpins genes (*i.e.*, homeologues of each other), whereas the homeologues of the serpins in groups 4-11 were not up-regulated by a stress and expressed in the grain.

** Bg = Blumeria graminis (powdery mildew).

Fg = *Fusarium* graminearum (*Fusarium* head blight).

Fp = *Fusarium* pseudograminearum (*Fusarium* crown rot).

Ps = Puccinia striiformis (Stripe rust).

Zt = Zymoseptoria tritici (Septoria tritici blotch).

- = no significant fold change by stress, or no expression (<0.5 TPM) in the grain.

**Figure 6:**
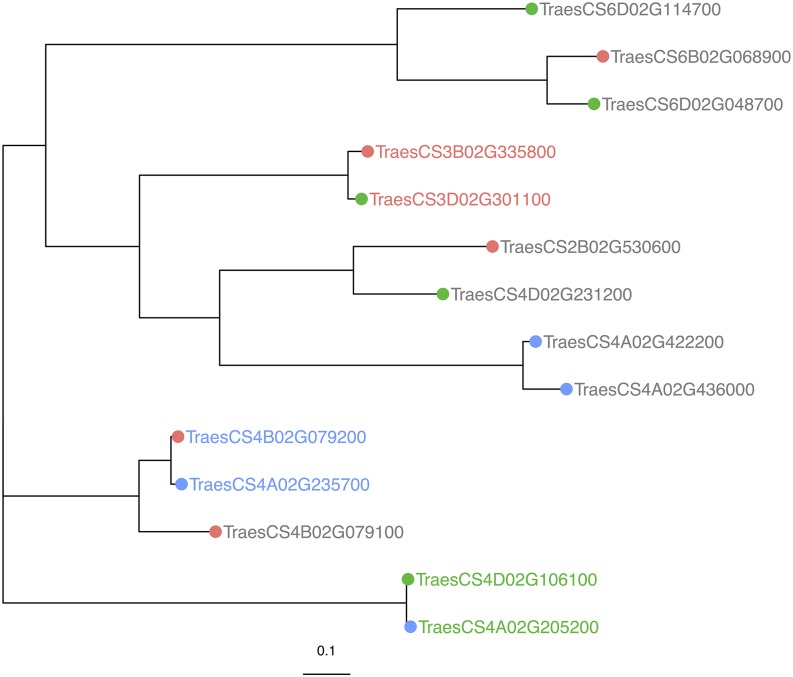
A neighbor-joining phylogenetic tree of 14 serpin genes that are expressed in the wheat grain and are disease responsive. Within this set of pleiotropic genes, three pairs were homeologues of each other, shown by the color of the label (genes of the same label color are homeologues). These 14 genes also grouped into one of 4 groups of paralogous genes. Tip point color represent these groupings, *i.e.*, genes with the same tip point color belong to the same group of paralogues (genes that are descended from the same ancestral gene). The scale bar indicates amino acid substitutions per site.

The 14 pleiotropic genes fell into one of four groups, where members of each group were paralogues of each other (*i.e.*, descended from the same ancestral gene) (Figure 6), and six of these were grouped into three sets of homeologues (these homeologous sets were previously described in Table 3). The homeologues TraesCS3B01G335800 and TraesCS3D01G301100 had similar expression patterns: both homeologues were up-regulated by *F. graminearum* and expressed in the endosperm and seed coat. The second homeologous group, TraesCS4D01G106100 and TraesCS4A01G205200, do not have similar biotic-stress expression profiles; TraesCS4D01G106100 is up-regulated by *F. graminearum* and TraesCS4A01G205200 by Flg22, and both genes are expressed in the endosperm and spike. The third group, homeologous serpins TraesCS4A01G235700 and TraesCS4B01G079200, are both expressed in all tissues of the developing grain. TraesCS4A01G235700 is down-regulated by *B. graminis*, *P. striiformis*, and upregulated by *Z. tritici*, while TraesCS4B01G079200 is down-regulated by *P. striiformis* only.

## Discussion

The serpin gene family has gained attention in wheat and barley for its role in cereal grain development (Østergaard *et al.* 2004; Roberts *et al.* 2003; Roberts and Hejgaard 2008). In addition, serpins have also been implicated in the disease response in soybean, maize, *Arabidopsis*, tomato, barley and wheat (Solomon *et al.* 1999; Erb *et al.* 2009; Laluk and Mengiste 2011; Jashni *et al.* 2015; Fluhr *et al.* 2012; Bhattacharjee *et al.* 2015; Bhattacharjee *et al.* 2017; Pekkarinen *et al.* 2007; Paper *et al.* 2007; Mirzadi Gohari *et al.* 2015; Lema Asqui *et al.* 2018; Li *et al.* 2018b). This is the first genome-wide characterization and expression analysis of this agronomically important gene family in hexaploid wheat. Using a variety of publicly-available genetic resources (Appels *et al.* 2018; Ramírez-González *et al.* 2018; Borrill *et al.* 2016), we identifed 189 putative serpins in the wheat genome. Compared to the diploid grass species barley, rice and *Brachypodium*, wheat has an average of 2.8 times more serpin genes, as would be expected given the hexaploid nature of the wheat genome. The reference annotations for *A. thaliana* and rice have been updated since the serpin analysis conducted by Fluhr *et al.* (2012), and thus we found higher numbers of serpins in these plants compared to the older analysis, based on orthologous gene analysis using EnsemblPlants. The serpin genes were distributed unevenly across the wheat genome, as has been observed for other gene families (*e.g.*, NAC: Borrill *et al.* 2017).

Several steps were taken to ensure the highest-quality phylogenetic tree was extracted from the wheat sequences. The phylogenetic tree represents the data well, as evidenced by high bootstrap branch support. This gene family is well conserved, indicating some level of functional maintenance of the gene family by natural selection. As is to be expected in a hexaploid species, serpin genes generally clustered in homeologous triads, indicating that these genes evolved in the ancestral grass of the three wheat genome donors. The exceptions to this were 24 serpins that had no homeologues. All but one of these genes had paralogues, which in all cases were on a chromosome of the same genome, and in many cases were adjacent tandem duplicated genes. This suggests that unlike the homeologous genes, these tandem duplications arose post-divergence of the wheat genome ancestors. Gene duplication can lead to functional redundancy and a reduction in selection pressure on a gene, allowing the accumulation of consequence-free mutations, and such paralogous genes are commonly found throughout the wheat genome (Glover *et al.* 2015).

While we can infer from the phylogenetic tree information about the evolution of the serpin gene family and relationships between genes, the infancy of the wheat gene annotation reference and the lack of empirical annotation (Appels *et al.* 2018) means that we cannot ignore the possibility of an incomplete picture of the serpin gene family, or incorrect assignment of a gene to a particular chromosome. While this is unlikely, given that the wheat reference sequence was generated from sequencing flow-sorted chromosomes (IWGSC 2014), there are serpins that cluster within a clade populated entirely by genes from a different group of chromosomes, as seen in Figure 1. In these cases, there is potential for incorrect chromosome assignment, as sequence homology tells us that the genes are more similar to members of a different chromosomal group than members of their own. Phylogenetics may thus help to resolve ambiguity in the chromosomal assignment of genes. Also, based on clustering of the four serpins that are currently assigned to an ‘unknown’ chromosome group with their closest homologs, it may be possible to rudimentarily deduce the previously unknown chromosomal assignment of these genes.

*Fusarium* head blight, *Fusarium* crown rot, stripe rust and Septoria tritici blotch are major wheat diseases that impose economically significant restraints on global grain production. Out of the 189 serpins found, 20 genes were responsive to PAMP elicitors (chitin or Flg22), or one or more of *F. graminearum*, *F. pseudograminearum*, *P. striiformis*, and *Z. tritici*, the causal agents of the aforementioned diseases. Chitin and Flg22 elicit PAMP-triggered immunity (PTI) in plants (Jones and Dangl 2006). PAMPs are recognized by Pattern Recognition Receptor (PRR) genes. This response can occur within the first hour of contact and, in *Arabidopsis*, has been reported to up-regulate the expression of three serpin/serpin-like genes (Zipfel *et al.* 2004). The PAMP-responsive serpins may therefore be involved in PTI – an early stage of defense against pathogen attack. Interestingly, none of the PAMP-responsive serpin genes were also responsive to the direct application of a fungal pathogen, at least at the time points analyzed. PAMP triggered immunity can be suppressed by successful pathogens and it is possible that the up-regulation of PAMP responsive serpins is halted or repressed by the effectors that the invading fungi can secrete. The disease responsive serpins are therefore likely to have evolved more recently, in response to fungal effectors, contributing to the more rapidly evolving effector-triggered immunity (ETI) branch of plant immunity.

The disease-responsive serpin proteins were distributed throughout the phylogenetic tree, and expression patterns did not cluster within clades of the tree. This pattern suggests that their role in disease response evolved randomly and independently of their ancestry, fitting with the features of ETI. However, the *Fusarium*-responsive serpins do show some evidence of non-random distribution; five of the *Fusarium* responsive serpins are found within one clade, which contains two pairs of homologous *Fusarium*-responsive serpins; a paralogous pair and a homeologous pair. The shared functionality of these homologs suggests a more ancient evolution of function compared to the more phylogenetically dispersed proteins. In the case of the paralogous *Fusarium*-responsive serpins on chromosome 4A, they are physically close together and share 80% protein sequence identity. As they share a function that their homeologues do not, it is likely that the function of these genes, and their duplication, occurred post divergence of the ancestral grass species, uniquely in the A-genome ancestor: *Triticum urartu*. The RCL amino acid sequence and length determines the inhibitory activity of a serpin (Lawrence *et al.* 1994; Zhou *et al.* 2001, Cohen *et al.*, 2019) and the RCL was identical between the *Fusarium*-responsive serpins on chromosome 4A, but variable with their evolutionary nearest neighbors, a homeologous pair from the group 2 chromosomes that were not responsive to disease. As these protein sequences are predicted from gene models, this striking variation warrants further exploration, especially if the sequence of the RCL can shed light on the disease response, or lack thereof, of these genes.

The homeologous *Fusarium*-responsive genes in this phylogenetic clade are on chromosomes 3B and 3D, but the 3A homeologue of these genes is not responsive to the pathogen. Functional redundancy in homeologous genes relaxes the selection pressure on the genes and can ultimately allow one member of a homeologous group to lose or diversify its function, without fatality to the plant (Osborn *et al.* 2003). Although the RNAseq data used in this study covered a range of cultivars and timepoints, cultivar specificity and genotype-by-environment interaction is an important feature of plant disease; especially within the ETI branch of plant immunity (Jones and Dangl 2006). Therefore, it is possible that given the correct combination of host cultivar, fungal isolate, environmental conditions, or timepoint, transcriptional activation of the A genome copy of this gene may be observed in response to *Fusarium* disease.

In accordance with Roberts *et al.* (2003), who observed an abundance of serpins in the endosperm of barley, the majority of the disease responsive serpins were upregulated in wheat heads in response to *F. graminearum* infection. Localization of serpins in the grain implies their defensive function of protecting the developing seed from protease degradation (Bao *et al.* 2018). If already active in the developing grain of wheat and responsive to fungal effector molecules, serpin proteins make ideal candidates for study with respect to disease resistance and grain yield.

The majority of studies focusing on serpin genes in cereals have reported the role of serpin proteins in the grain (Østergaard *et al.* 2000; Roberts *et al.* 2003; Østergaard *et al.* 2004; Francis *et al.* 2012). Unsurprisingly, therefore, we observed a large number (55) of serpin genes expressed in the grain or head of wheat at various growth stages. The SERPIN-Z family is a group of six serpins found on the group 5 chromosomes. These serpin proteins have previously been partially sequenced (Rosenkrands *et al.* 1994; Østergaard *et al.*, 2000), and were found to have glutamine-rich RCLs, resembling prolamine grain storage proteins (Østergaard *et al.* 2000). Furthermore, five of these serpins (WSZ1a, WSZ1b, WSZ1c, WSZ2a and WSZ2b) were confirmed to inhibit the serine proteases chymotrypsin and cathepsin G (Østergaard *et al.* 2000). We identified this subclade of serpins and assigned the previously used serpin nomenclature to the IWGSC protein annotation codes and suggest that the previously named ‘WSZ’ protein by Rosenkrands *et al.* (1994) is, in fact, WSZ2c. This comparison validates our discovery of these genes and facilitates assigning annotation and description to IWGSC gene ID codes.

In addition to the six high-confidence SERPIN-Z proteins, we identified three more proteins with high sequence homology to the SERPIN-Z proteins. These three new serpins are ‘low-confidence’ annotations, *i.e.*, they are less supported by homology to proteins from other species than the high-confidence annotations (Appels *et al.* 2018). The reference sequences for these low-confidence proteins are either truncated or incomplete, so it may be possible that they are simply erroneous, duplicated partial annotations of the six SERPIN-Z proteins. However, they are expressed in the grain tissues, and therefore may be true genes, unless their sequence similarity to other serpin-Z genes caused false alignment during the RNA read mapping stage (described by Borrill *et al.* 2016; Ramírez-González *et al.* 2018). The low-confidence genes are located on the same chromosome, 5B. The B-genome is postulated to derive from paraphyletic origins and experienced high genome plasticity post-polyploidisation compared to the A and D genomes (El Baidouri *et al.* 2017). Genome plasticity facilitates genome re-arrangements, tandem duplications and transposable elements and can result in transcriptional restructuring (Leitch and Leitch 2008). Therefore, it is possible that these genes are unique to wheat and are restricted to the B-genome. Independent validation of these genes is required to determine their status but, given their role in the grain of wheat, these genes may make for important and previously undiscovered targets for accelerating genetic gains in wheat yields.

We found 14 serpin genes with apparent pleiotropic activity; expressed in the developing grain of wheat and also responsive to disease. These 14 genes could be grouped into four paralogous groups and three homeologous groups. The genes are functionally conserved in terms of their expression in the developing grain, but their expression profiles across diseases are inconsistent, indicating that their role in disease response evolved much more recently than in grain development. The recent evolution of disease response, and the uneven expression of these genes across their homeologues, is consistent with the rapid evolution of ETI. Seven of the pleiotropic serpin genes were upregulated by the wheat head disease *F. graminearum*. The *F. graminearum* response of these genes may be due to their prior localization in the developing grain; serpin proteins may have evolved to recognize and inhibit protease enzymes from invading *F. graminearum*. Five of the pleiotropic serpins, were down-regulated by *B. graminis*, *P. striiformis* and *F. pseudograminearum*. Interestingly, *B. graminis* and *P. striiformis* are biotrophic pathogens that actively feed on the host during infection, and both are known to secrete serine proteases as part of their metabolic activity (Both *et al.* 2005; Xia *et al.* 2017). These pathogenic fungi may have evolved to overcome the anti-metabolic reaction of the plant by suppressing transcription of these inhibitory serpins, and consequently securing the success of their own secreted proteases. Investigating the transcription of these serpins, especially those repressed as part of the metabolic activity of the invading pathogen, may shine light on our understanding of more complicated pathogens, such as the controversially-titled hemibiotroph *Z. tritici*.

## Conclusions

This is the first whole-genome report of the serpin gene family of wheat, in particular in relation to their role in the disease response. Based on the expression profiles of the serpins across the available RNAseq studies, it is evident that this family of genes make interesting targets for characterization, especially in response to FHB disease of wheat. Our future work will focus on validating and functionally characterizing the role of the most interesting of these genes and identifying the interacting proteases that are inhibited by these proteins.
